# Evaluation of a novel PXR‐knockout in HepaRG^™^ cells

**DOI:** 10.1002/prp2.264

**Published:** 2016-09-21

**Authors:** Beth Williamson, Mathias Lorbeer, Michael D. Mitchell, Timothy G. Brayman, Robert J. Riley

**Affiliations:** ^1^ Evotec (UK) Ltd114 Innovation DriveAbingdonOxfordshireOX14 4RZUnited Kingdom; ^2^ Science and Technology CentreSigma‐AldrichSt. LouisMissouriUSA

**Keywords:** DDIs, human PXR, induction, knockout

## Abstract

The nuclear pregnane X receptor (PXR) regulates the expression of genes involved in the metabolism, hepatobiliary disposition, and toxicity of drugs and endogenous compounds. PXR is a promiscuous nuclear hormone receptor (NHR) with significant ligand and DNA‐binding crosstalk with the constitutive androstane receptor (CAR); hence, defining the precise role of PXR in gene regulation is challenging. Here, utilising a novel PXR‐knockout (KO) HepaRG cell line, real‐time PCR analysis was conducted to determine PXR involvement for a range of inducers. The selective PXR agonist rifampicin, a selective CAR activator, 6‐(4‐chlorophenyl)imidazo[2,1‐b][1,3]thiazole‐5‐carbaldehyde O‐(3,4‐dichlorobenzyl)oxime (CITCO), and dual activators of CAR and PXR including phenobarbital (PB) were analyzed. HepaRG control cells (5F clone) were responsive to prototypical inducers of CYP2B6 and CYP3A4. No response was observed in the PXR‐KO cells treated with rifampicin. Induction of CYP3A4 by PB, artemisinin, and phenytoin was also much reduced in PXR‐KO cells, while the response to CITCO was maintained. This finding is in agreement with the abolition of functional PXR expression. The apparent EC_50_ values for PB were in agreement between the cell lines; however, CITCO was ~threefold (0.3 *μ*mol/L vs. 1 *μ*mol/L) lower in the PXR‐KO cells compared with the 5F cells for CYP2B6 induction. Results presented support the application of the novel PXR‐KO cells in the definitive assignment of PXR‐mediated *CYP2B6* and *CYP3A4* induction. Utilization of such cell lines will allow advancement in composing structure activity relationships rather than relying predominantly on pharmacological manipulations and provide in‐depth mechanistic evaluation.

**Abbreviations**
C_(*t*)_: comparative threshold
CAR: constitutive androstane receptor
CHPM: cryopreserved plating hepatocyte media
CHRM: cryopreserved hepatocyte recovery media
CITCO: 6‐(4‐Chlorophenyl)imidazo[2,1‐b][1,3]thiazole‐5‐carbaldehyde O‐(3,4‐dichlorobenzyl)oxime
CYP: cytochrome P450
DDI: drug–drug interaction
DMSO: dimethyl sulfoxide
E_max_: maximum observed effect
EC_50_: concentration at half maximum induction
GAPDH: glyceraldehyde‐3‐phosphate dehydrogenase
KO: knockout
LBD: ligand‐binding domain
NCEs: new chemical entities
NHR: nuclear hormone receptor
NR: not reported
PB: phenobarbital
PCR: polymerase chain reaction
PHH: primary human hepatocyte
PK: pharmacokinetics
PXR: pregnane X receptor
RT: reverse transcription
WT: wild type
ZFN: zinc finger nuclease

## Introduction

Induction of cytochrome P450 (CYP) enzymes can result in significant drug–drug interactions (DDIs) via suboptimal drug exposure and reduced efficacy (Chu et al. 2009) and/or differential metabolism, for example, bioactivation to reactive species (Sinz et al. 2008). However, CYP induction analysis has only recently become part of the routine drug discovery process (Smith 2000; Riley and Wilson 2015). Consequently, high throughput screens early in the discovery cascade have become increasingly common. In a recent analysis of 309 compounds, up to 33% were found to be CYP inducers (Badolo et al. 2015).

The pregnane X receptor (PXR) is a nuclear hormone receptor (NHR) which regulates the expression of genes involved in the hepatobiliary disposition and toxicity of drugs and endogenous compounds (Hariparsad et al. 2008). Upregulation of *CYP3A4* gene transcription via PXR is a mechanism for which clinically significant DDIs are observed (Shou et al. 2008). In contrast to most NHRs, the ligand‐binding domain (LBD) of PXR is not conserved across species, with <80% homology between mammals (Iyer et al. 2006), making interpretations from animal studies more complex. Furthermore, PXR is a promiscuous NHR with significant ligand‐ and DNA‐binding crosstalk with constitutive androstane receptor (CAR) (Iyer et al. 2006). Treatment with a non‐selective PXR/CAR agonist results in a coordinated and highly efficient response by the NHRs through the concerted activation of mutual target genes (Chen et al. 2005). For example, phenobarbital (PB) induces *CYP3A4* and *CYP2B6* through both PXR and CAR. While *CYP2B6* is primarily a CAR target, it is also induced by the selective PXR agonist rifampicin; hence defining the precise role of PXR in gene regulation is challenging.

While primary human hepatocytes (PHHs) are considered the gold standard model for induction analysis, their availability, short life span, cost, and large interindividual variability limit their use in drug discovery (Madan et al. 1999; Hewitt et al. 2007; Godoy et al. 2013). The search for simpler in vitro models and robust alternative cell sources to determine the induction potential of new chemical entities (NCEs) continues (Godoy et al. 2013). The NHR PXR and CAR gene reporter assays can be useful high throughput screens for PXR/CAR activators (Hariparsad et al. 2008). However, these assays are susceptible to false negatives and should be used with caution. Due to a lack of in vitro assays, CAR analysis is not yet part of the traditional screening cascade for most companies. Alternatively, spliced transcripts also complicate CAR analysis. For example, low basal activity is observed in the CAR3 transcript, but the variant is significantly activated by direct and indirect CAR ligands (Gupta et al. 2008). While several crystal structures of PXR and its LBD have been generated, established structure activity relationships (SAR) which predict well and attenuate PXR activation remain elusive (Chu et al. 2009). Pharmacophore models and in silico docking approaches have provided some guidance for drug design, but are primarily used as ranking/filtering methods (Ekins and Erickson 2002; Gao et al. 2007; Ung et al. 2007).

The inducible hepatic cell line, Fa2N‐4, developed by MultiCell Technologies (Lincoln, RI) showed some early promise (Mills et al. 2004). However, due to a lack of CAR expression the cell line is primarily used to identify PXR activators (Ripp et al. 2006; McGinnity et al. 2009). Basal gene expression of the hepatic uptake transporters is significantly lower in Fa2N‐4 cells compared to PHHs, thus Fa2N‐4 cells will significantly underestimate the induction potential for some compounds (Tirona and Kim 2002; Templeton et al. 2011). The human colon carcinoma cell line, LS180, is widely used to predict the intestinal induction potential of NCEs, but is similarly limited by its lack of functional CAR expression (Gupta et al. 2008). HepaRG cells are now recognized as a suitable alternative to PHHs since they exhibit hepatocyte‐like function and morphology as well as expressing specific hepatic drug disposition genes (Lubberstedt et al. 2011). Mesenchymal stem cells may be a suitable alternative to PHHs (Sa‐Ngiamsuntorn et al. 2011). Cell proliferation is maintained for 6 months in addition to hepatocyte‐like morphology and phenotype. While the basal expression and activity of some CYPs is lower than in PHHs (Grime et al. 2010; Zanelli et al. 2012), the cells prove to be a sensitive model as they respond to prototypical inducers. However, the potential impact and application of this model has yet to be realized fully due to the lack of NHR understanding in these cells.

Previous work has involved the generation of NHR knockout (KO) HepaRG cells through exploitation of the error‐prone nonhomologous end‐joining (NHEJ) pathway (Brayman et al. 2014). Utilizing zinc finger nucleases (ZFNs), targeted double strand breaks of DNA are generated. In some cells, the subsequent DNA repair process results in insertions and/or deletions of the target gene resulting in inactivity. These genetically engineered cells, 5F and PXR‐KO, retain basal CYP enzyme activity, uridine diphosphate glucuronosyltransferase activity, and drug transport activity, in addition to hepatic‐like morphology (Brayman et al. 2014); clearly demonstrating selective PXR‐KO and no associated off‐target effects.

Here, utilizing these novel zinc finger nuclease (ZFN) targeted PXR‐KO HepaRG cell line and the parent HepaRG cell line 5F, the contribution of PXR following treatment with known CYP3A4 and CYP2B6 inducers was evaluated.

## Materials and methods

### Materials

5F and PXR‐KO HepaRG cells used were supplied by Sigma‐Aldrich (St. Louis, USA). Three Lots of human hepatocytes were used in this study, Lots Hu1455 (D1), Hu1601 (D2), and Hu8132 (D3) (Life Technologies, Paisley, UK). HepaRG wild type (WT), cryopreserved hepatocyte recovery media (CHRM), cryopreserved plating hepatocyte media (CHPM), collagen‐1‐coated 96‐well plates, reverse transcription reagents, universal master mix, HepaRG thawing and maintenance medium, HepaRG serum‐free induction medium, and Taqman gene expression assays (Table 1) were purchased from Life Technologies. Recovery, maintenance, and serum‐free induction media supplements were purchased from Caltag Medsystems Ltd. (Buckingham, U.K.). SV96 total RNA isolation system was purchased from Promega (Southampton, U.K.). ZR‐96 Quick‐RNA was obtained from ZymoResearch (CA, USA). Mirus Trans‐It mRNA Transfection reagent were purchased from Mirus Bio (Madison, WI). All other materials were purchased from Sigma‐Aldrich (Dorset, U.K.).

**Table 1:** List of genes analyzed using real‐time PCR

Gene	Description	Reference sequence	Assay ID
*CYP3A4*	Cytochrome P450, family 3, subfamily A, polypeptide 4	NM_001202855.2	Hs_00604506_m1
*CYP2B6*	Cytochrome P450, family 2, subfamily B, polypeptide 6	NM_000767.4	Hs_04183483_g1
*GAPDH*	Glyceraldehyde‐3‐phosphate dehydrogenase	NM_001256799.1	Hs_02758991_g1

Assay ID is the reference number and gene ID is the NCBI reference number. Dye – FAM: 6‐fluorescein amidite.

### KO creation

The HepaRG cell line was modified by delivering ZFN pairs by transfection with Mirus Trans‐It mRNA Transfection reagent. Single viable cells were sorted by flow cytometry and resultant colonies were tested for mutations by amplifying genomic DNA using ZFN Cel‐1 primers, followed by PCR on target regions using HEX/FAM‐labeled nested primers. DNA sequence of the target regions was analyzed to confirm gene disruption through deletion or insertion. Clones containing gene disruption in both alleles were expanded for functional KO analysis. The final PXR‐ and CAR‐KO clones were selected based on cell morphology, growth characteristics, and lack of response to the CYP3A4 inducer rifampicin and the CYP2B6 inducer 6‐(4‐chlorophenyl)imidazo[2,1‐b][1,3]thiazole‐5‐carbaldehyde O‐(3,4‐dichlorobenzyl)oxime (CITCO). A second KO clone (5F) was generated in the same manner using a ZFN pair targeted against a noncritical portion of the genome for use as a control cell line.

### Cryopreserved human hepatocyte cell culture

Cryopreserved male human hepatocytes were thawed in CHRM and plated into a 96‐well collagen‐1‐coated plate at a density of 7.5 × 10^4^ viable cells in 200 *μ*L of CHPM. Trypan blue exclusion was used to determine cell viability with a cut‐off of 85% viability. The cells were incubated at 37°C in a humidified incubator with 5% CO_2_. Plating medium was replaced with maintenance medium following 4 h incubation and then maintained overnight before 48 h treatment with test compound. Test compounds were freshly dissolved in dimethyl sulfoxide (DMSO) and then culture medium to achieve a final DMSO concentration of 0.1%. A vehicle control of medium with 0.1% DMSO was included in all studies.

The concentration range for each compound was selected to provide an initial estimate of EC_50_ based on evaluation of available literature as documented previously (McGinnity et al. 2009).

### HepaRG cell incubation

HepaRG cells were thawed in thawing and maintenance medium and plated into 96‐well collagen‐1‐coated plates at a volume of 200 *μ*L/well. The cells were incubated at 37°C in a humidified incubator with 5% CO_2_ for 72 h. Test compounds were freshly dissolved in DMSO and then serum‐free induction medium to achieve a final DMSO concentration of 0.1% every 24 h.

## 5F, CAR‐KO, and PXR‐KO HepaRG cells incubation

5F, CAR‐KO, and PXR‐KO HepaRG cells were thawed in recovery medium and plated into 96‐well collagen‐1‐coated plates at a volume of 200 *μ*L/well. The cells were incubated at 37°C in a humidified incubator with 5% CO_2_ for 48 h. The recovery medium was replaced with maintenance medium and procedure repeated every Monday, Wednesday, and Friday for 18 days. On day 18, the maintenance medium was replaced with pre‐incubation medium for 72 h. On day 21, the cells were incubated with test compounds in serum‐free induction medium for 48 h. Test compounds were freshly dissolved in DMSO and then serum‐free induction medium to achieve a final DMSO concentration of 0.1% every 24 h. For enzyme induction assays, cells were thawed into recovery medium and plated into 24‐well plates at a volume of 1 mL/well. Culture conditions were as described above.

### mRNA extraction and reverse transcription

mRNA was extracted from the cell monolayers using the SV96 total RNA isolation system according to the manufacturer's instructions. Reverse transcription of mRNA to cDNA was completed using Taqman reverse transcription (RT) assay. RT mixtures were prepared according to the manufacturer's instructions; 25 *μ*L reactions consisted of: 10X Taqman RT buffer, MgCl_2_ (5.49 mmol/L), reverse transcriptase (1 *μ*mol/L), RNA (2 *μ*g), dNTP (50 *μ*mol/L), oligo‐d(T) (2.5 *μ*mol/L), and RNase inhibitor (1 *μ*mol/L). An Agilent Mx3005P thermocycler was used to run a thermal cycle of: 10 min at 25°C, 30 min at 37°C, 5 min at 95°C, and a hold phase at 4°C.

### Quantitative real‐time PCR gene expression analysis

An Agilent Mx3005P thermocycler was used to determine the gene expression of selected genes. Real‐time PCR solutions were prepared as described by the manufacturer. Each reaction contained 12.5 *μ*L volume. Table 1 details the assays (Life Technologies) used for each gene with its ID. PCR conditions were 15 min at 95°C (to activate polymerase, denature cDNA, and initiate PCR) followed by 40 cycles of 15 sec at 94°C (denaturation), and 60 sec at 60°C (annealing/extension of the product). Fluorescence was measured at the end of each cycle.

No template controls were completed in duplicate to ensure no contamination, specific amplification, and maximum amplification, respectively. *Glyceraldehyde‐3‐phosphate dehydrogenase* (*GAPDH*) was used as a housekeeping gene (C_(*t*)_ values were consistent in every sample). To ensure only gene amplification was measured, the C_(*t*)_ was set to ignore any aberrant fluorescence such as that from primer–dimer formation.

### Induction activity data

P450 enzyme activity was assessed by incubating whole‐cell monolayers for 2 h at 37°C using 100 *μ*mol/L bupropion (CYP2B6) in unsupplemented Williams E media with a final concentration of 0.1% DMSO. Reactions were quenched by removing supernatants and diluting 1:2 into ice‐cold acetonitrile. Samples were stored at −20°C until LC‐MS/MS analysis was completed.

### Activity analysis using LC‐MS/MS

LC‐MS/MS analysis utilized an API‐4000 Q Trap mass spectrometer with a Turbo V atmospheric pressure electrospray ionization source (AB SCIEX, Framingham, MA). Samples (40 *μ*L) were injected onto a Fortis C18 column (3 × 50 mm, 5 *μ*m) and eluted by a mobile phase gradient specific for each test article (mobile phase A: 0.1% formic acid in water; mobile phase B: 0.1% formic acid in acetonitrile). Flow rate was 0.5 mL/min. MS conditions: positive or negative ionization mode (4.5 kV spray voltage); source temperature of 450°C with multiple reaction monitoring specific for each analyte and internal standard parent–product ion pairs. Peak areas of analyte and internal standard and resulting ratios were quantified using Analyst 1.5.2 (AB SCIEX, Framingham, MA).

### Data analysis

Gene expression data were compared to an average of *GAPDH* and normalized to the control sample using the comparative threshold cycle (C_(*t*)_) method (C_*t*_ = 2^−∆∆C(*t*)^). Pearson correlation coefficient (GraphPad Prism 6, CA, USA) was used to compare the EC_50_ values generated by the 5F and PXR‐KO HepaRG cells. Where possible data were fitted to a sigmoidal *E*_max_ model (WinNonLin Phoenix, Pharsight, 6.3.0.395). All human hepatocyte data are the average of duplicate experiments and three compound concentrations. HepaRG WT are the average of triplicate experiments completed in duplicate. HepaRG 5F and HepaRG PXR‐KO data are the average of duplicate experiments and duplicate replicates of each compound concentration (six concentrations).

## Results

### Basal gene expression

Basal gene expression was assessed in the hepatic cell lines and primary human hepatocytes. Data were compared to human hepatocyte donor 1 (D1; selected at random). In agreement with earlier reports (Rogue et al., 2012) basal expression of *CYP3A4* and *CYP2B6* varied considerably between human donors (30–100% of D1). Following the culture of each cell line, compared to PHH (D1), *CYP3A4* basal expression was lower in HepaRG WT (75% of control) and 5F cells (60% of control). *CYP2B6* was also expressed to a greater extent in all three PHH donors compared to the HepaRG WT (70% of control) and 5F (68% of control) cells.

### Comparison of CYP induction

**Figure 1:**
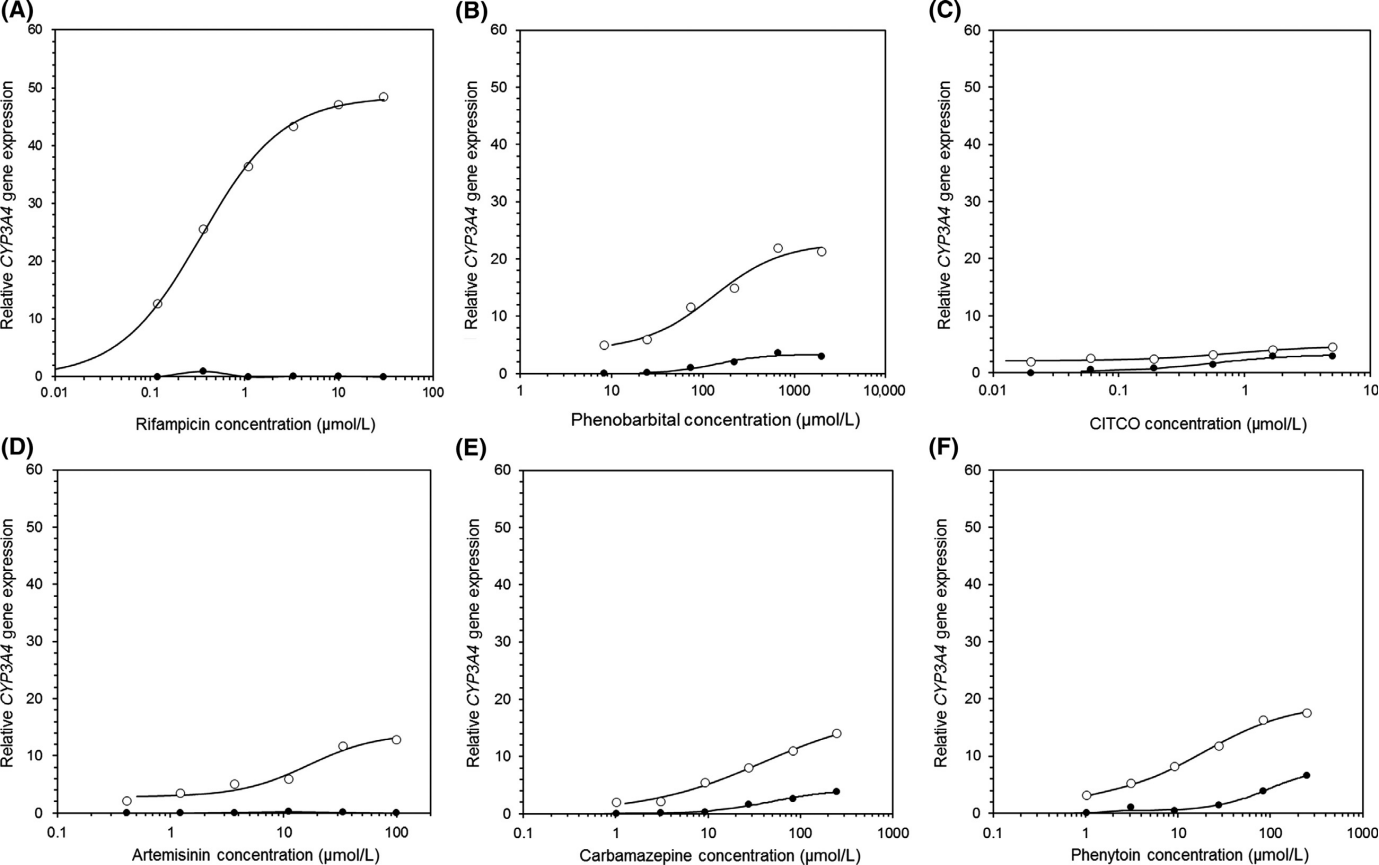
Dose–response curves of *CYP3A4* gene expression in 5F (o) and PXR‐KO (•) cells following treatment with known inducers. PXR‐KO, pregnane X receptor‐knockout.

**Figure 2:**
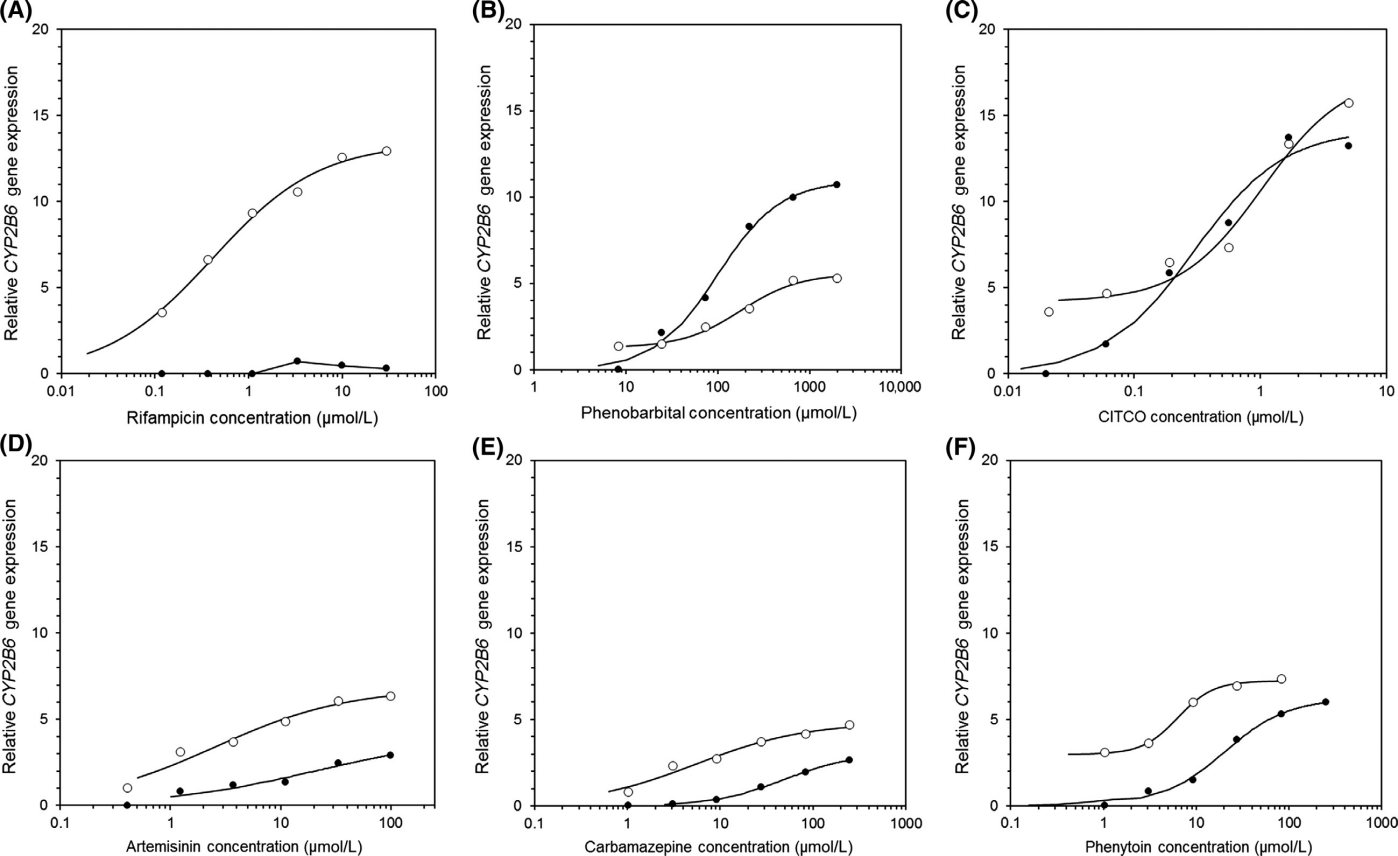
Dose–response curves of *CYP2B6* gene expression in 5F (o) and PXR‐KO (•) cells following treatment with known inducers. PXR‐KO, pregnane X receptor‐knockout.

All compounds tested displayed no cytotoxic effects in any cell type, therefore all data were included in the analysis. To determine whether the 5F and PXR‐KO cells could be used as a predictive mechanistic model of CYP induction, seven known CYP3A4 and CYP2B6 inducers were selected for comparison of concentration‐dependent effects (Figs. 1, 2). Before defining any mechanistic effects, the 5F cells were compared to HepaRG WT cells and PHH (Tables 2, 3). Replication of known inducers in human hepatocytes provided confidence that the results generated were in agreement with those described in the literature and provided a benchmark for comparison of the prospective cell lines.

**Table 2:** *E*_max_ and EC_50_ (*μ*mol/L) values for induction of *CYP3A4* mRNA for compounds tested in 5F cells, HepaRG wild‐type cells, and three primary human hepatocyte donors

Compound					Primary human hepatocyte donors					
	5F		HepaRG		D1		D2		D3	
	*E*_max_	EC_50_	*E*_max_	EC_50_	*E*_max_	EC_50_	*E*_max_	EC_50_	*E*_max_	EC_50_
Rifampicin	46.1, 51.7	0.5, 0.3	79.5 ± 9.0	0.4 ± 0.2	228.3	1.1	80.8	12.5	226.4	2.1
Phenobarbital	17.3, 28.1	108.4, 166.2	110.0 ± 12.8	79.7 ± 23.6	21.1	250.6	13.3	365.6	81.8	176.9
Artemisinin	15.6, 20.8	17.5, 11.3	45.1 ± 3.3	20.5 ± 8.1	4.5	50.0	NE	NA	30.3	>50.0
CITCO	6.4, 7.1	1.9, 2.0	21.1 ± 4.6	1.5 ± 0.1	3.5	0.3	NE	NA	2.3	0.4
Carbamazepine	29.3, 21.6	37.4, 42.1	33.5 ± 12.6	24.0 ± 12.2	NR		NR		NR	
Phenytoin	16.0, 20.8	18.1, 19.9	89.6 ± 8.1	42.6 ± 6.6	NR		NR		NR	

*E*_max_ and EC_50_ are expressed for each individual experiment for 5F and primary human hepatocytes (D1, D2, D3). *E*_max_ ± SD and EC_50_ ± SD are expressed for HepaRG cells. All data were calculated using GraphPad as described under Materials and methods section.

CITCO, 6‐(4‐Chlorophenyl)imidazo[2,1‐b][1,3]thiazole‐5‐carbaldehyde O‐(3,4‐dichlorobenzyl)oxime; NE, no effect observed; relative gene expression fold change <2; NA, not applicable; NR, not reported; abbreviated concentration range selected did not afford parameter estimate.

**Table 3:** *E*_max_ and EC_50_ (*μ*mol/L) values for induction of *CYP2B6* mRNA for compounds tested in 5F cells, HepaRG cells, and primary human hepatocytes

Compound					Primary human hepatocyte donors					
	5F		HepaRG		D1		D2		D3	
	*E*_max_	EC_50_	*E*_max_	EC_50_	*E*_max_	EC_50_	*E*_max_	EC_50_	*E*_max_	EC_50_
Rifampicin	18.9, 12.9	1.7, 0.7	5.2 ± 2.9	0.3 ± 0.2	3.9	0.1	4.3	5.2	16.3	5.5
Phenobarbital	5.9, 5.5	145.3, 195.4	68.9 ± 47.3	170.3 ± 6.8	20.2	98.9	NE	NA	26.3	185.7
Artemisinin	6.7, 9.4	3.6, 9.8	11.1 ± 4.9	2.2 ± 0.6	NE	NA	NE	NA	2.5	0.4
CITCO	17.0, 22.5	1.0, 0.3	14.2 ± 7.4	0.03 ± 0.01	6.0	0.2	NE	NA	5.5	0.3
Carbamazepine	3.1, 7.4	8.4, 5.4	10.7 ± 4.3	12.0 ± 7.3	NR		NR		NR	
Phenytoin	7.3, 10.6	5.1, 7.8	14.2 ± 3.7	2.7 ± 1.7	NR		NR		NR	

*E*_max_ and EC_50_ are expressed for each individual experiment for 5F and primary human hepatocytes (D1, D2, D3). *E*_max_ ± SD and EC_50_ ± SD are expressed for HepaRG cells. All data were calculated using GraphPad as described under Materials and methods section.

CITO, 6‐(4‐Chlorophenyl)imidazo[2,1‐b][1,3]thiazole‐5‐carbaldehyde O‐(3,4‐dichlorobenzyl)oxime; NE, no effect observed; relative gene expression fold change <2; NA, not applicable; NR, not reported; abbreviated concentration range selected did not afford parameter estimate.

As expected, a large range was observed for *CYP3A4* maximum effective response (*E*_max_) between the PHH donors (Table 2). In contrast, the range for *CYP2B6 E*_max_ was in agreement for two of the three donors (Table 3). Donor 2 (D2) was particularly poor at responding to any CYP2B6 inducer and, in particular, selective CAR activators. As detailed in the Materials and methods section, test compounds were replaced daily to minimize compound turnover, hence it was not measured throughout the analysis. While there are views that compound turnover may impact the outcome, inclusion and application of the parameter are not completely understood (Honma et al. 2010).

The lack of observable *E*_max_ for phenytoin and carbamazepine in the PHHs using the concentrations selected for both CYPs hindered data interpretation as the dose–response could not be fitted to a sigmoidal *E*_max_ model (Tables 2, 3).

In keeping with the lower basal gene expression, the 5F cells generally had lower *E*_max_ values when compared to the HepaRG WT cells. However, EC_50_ values for each compound between the two hepatic cell lines were in good agreement for *CYP3A4* (*r* ^2^ = 0.84, *P* < 0.0097) and *CYP2B6* (*r* ^2^ = 0.98, *P* < 0.0001). Furthermore, data for each hepatic cell line agreed with the reference human hepatocyte donor, D1.

The selective activators rifampicin (PXR) (Lecluyse 2001b) and CITCO (CAR) (Maglich et al. 2003; Simonsson et al. 2006) were investigated as part of this test set of compounds. In addition, the dual PXR/CAR activators PB, artemisinin, carbamazepine, and phenytoin were also analyzed (Hariparsad et al. 2004; Wang et al. 2004; Trubetskoy et al. 2005; Bell and Michalopoulos 2006; Faucette et al. 2007). All 5F and PXR‐KO data are the average of two experiments completed in duplicate. Dose–response data obtained following treatment of the 5F and PXR‐KO cells with the known inducers were fitted to a sigmoidal *E*_max_ model and EC_50_ values generated were applicable.

5F cells were responsive to prototypical inducers of CYP3A4 (rifampicin) and CYP2B6 (CITCO). EC_50_ values of 0.3 *μ*mol/L (*CYP3A4*, rifampicin) and 0.5 *μ*mol/L (*CYP2B6*, CITCO) were broadly in agreement with those observed in the PHHs (Figs. 1, 2). The lack of functional PXR expression in the PXR‐KO cells was confirmed following rifampicin treatment, which resulted in no response for *CYP3A4* or *CYP2B6*.

Possible future application of the PXR‐KO cells was evident following treatment with dual PXR/CAR activators. For PB in the PXR‐KO cells, the EC_50_ (144 *μ*mol/L) and *E*_max_ (3.3) values of *CYP3A4* were greatly reduced in comparison to the other cell types (Fig. 1B, Table 2). Correspondingly, the EC_50_ and *E*_max_ values of D1 were 250 *μ*mol/L and 21, respectively; values obtained for the 5F and HepaRG WT cells were also within two‐fold of D1 (Table 2). The opposite trend was observed for *CYP2B6* in the PXR‐KO cells, following PB treatment. The dose–response had a higher *E*_max_ (11.8) and lower EC_50_ (98 *μ*mol/L) when compared to the 5F cells (Fig. 2B). These values were in agreement with those observed for D1 and HepaRG WT cells. Although not as pronounced, a similar two‐fold decrease in EC_50_ was observed upon CITCO treatment for *CYP2B6* (Fig. 2C).

**Figure 3:**
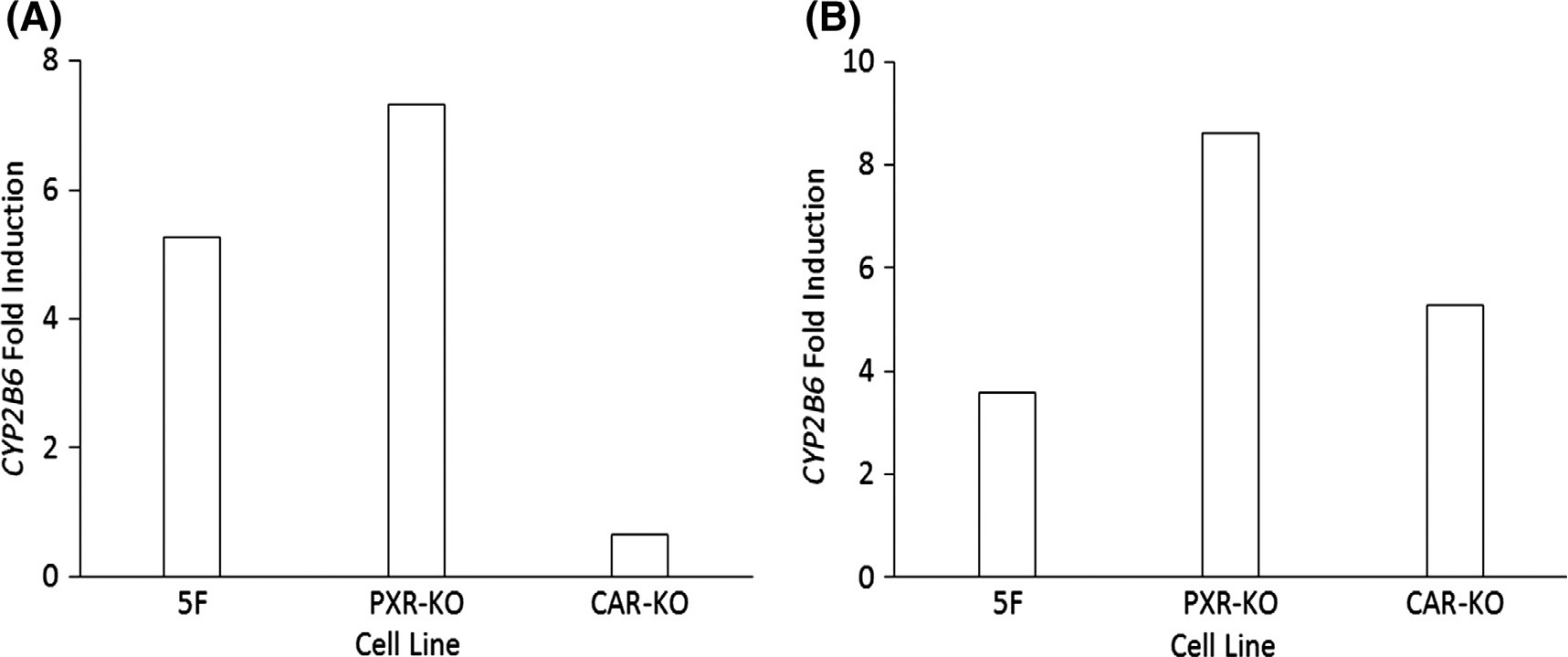
Induction response of CYP2B6 in NHR‐KO cells and 5F cells following treatment with 1.5 *μ*mol/L CITCO (A) and 258 *μ*mol/L phenobarbital (B) compared to untreated NHR‐KO and 5F cells. NHR, nuclear hormone receptor knockout.

mRNA data are in agreement with effects observed at the enzymatic level (Fig. 3). While basal activity levels of CYP2B6 between the 5F and PXR‐KO cells were similar, the CYP2B6‐induced activity response following CITCO (Fig. 3A) and PB (Fig. 3B) treatment was greater in the PXR‐KO cells. Furthermore, the same trend was not observed for CYP3A4. This finding could be attributed to compensatory upregulation of other genes as reported for HepaRG CAR‐KO cells (Li et al. 2015). Visual inspection of the *CYP2B6* concentration–response curves suggests a biphasic response for CITCO (Fig. 2C). Multiple EC_50_ values may be possible if the compound is able to bind with varying affinities to alternative targets on the NHR.

For the additional PXR/CAR activators, artemisinin, carbamazepine, and phenytoin, the *E*_max_ was lower in the PXR‐KO cells for *CYP2B6* and *CYP3A4* in comparison to the 5F cell line, HepaRG WT cells, and PHHs. Likewise, the EC_50_ values were increased due to a right shift of the dose–response curves.

These data supported the conclusions that functional PXR is not present in the PXR‐KO cells; however, the cells retain a functional CAR pathway allowing identification of selective PXR activators.

## Discussion

Although PXR is the main NHR through which induction DDIs (Moore et al. 2000; Sinz 2013) are mediated, it is difficult to assign the exact mechanism directly. Despite advances in generating hepatocyte‐like models to predict CYP induction and genetic modification of CAR in rodent models to define the NHR's role, mechanistic human prediction remains elusive. Given the complexity of the human system, it is not surprising that no single in vitro model is capable of replicating the observed in vivo effect. Emerging technologies comprise human liver slices, animal models, chimeric or humanized animal models however, as for PHHs, all are hindered by availability, cost, and lack of refined and optimized processes (Chu et al. 2009).

Here, utilizing a novel ZFN‐targeted PXR‐KO HepaRG cell line and the 5F HepaRG cell line, the contribution of PXR following treatment with known CYP3A4 and CYP2B6 inducers was determined. The in vitro induction potential of each compound was determned by gene expression with each concentration curve covering the relevant responsive range determined from previous work (McGinnity et al. 2009), up to maximal non‐cytotoxic concentrations.

With copious amounts of evidence throughout the literature, significant human donor variability in gene expression and activity of proteins involved in xenobiotic metabolism, including (not exclusively) CYP1A2, CYP2B6, CYP3A4, and UGTs, is well established (Lecluyse 2001a; Goyak et al. 2008; Yang et al. 2013). In addition to the donor and culture conditions, laboratory treatment, donor genotype, donor history, including medication use and disease status, can greatly influence the gene expression observed in vitro (Zhou et al. 2009; Guguen‐Guillouzo and Guillouzo 2010; Russmann et al. 2010). For example, steatotic livers have significantly variable CYP activities in comparison to healthy donors (Gomez‐Lechon et al. 2004). Indeed, results herein showed one donor performed poorly in our hands (Tables 2, 3), emphasizing laboratory and user conditions can impact significantly.

Advances in in vitro analysis aim to efficiently represent human donors as well as reducing the variability observed between laboratories and experiments. Recent work has demonstrated HepaRG variability between experiments is particularly low (Vermet et al. 2016), which is further confirmed with alternative HepaRG batches used herein. Similarly, E_max_ values between PHH donors varied considerably (Tables 2, 3), whereas a low interexperiment variability was observed for HepaRG cells.

Additional considerations include compound turnover throughout the incubation period as the extent of CYP induction is dependent on drug exposure. To minimize this effect compound is replaced daily. The consistent assessment, inclusion, and interpretation of compound turnover in such studies are still a matter of investigation and debate (Honma et al. 2010).

To determine whether the 5F HepaRG cells were initially a suitable model for induction, the basal gene expression of *CYP3A4* and *CYP2B6* was initially determined. Lower basal gene expression in HepaRG WT and 5F cells found were in agreement with previous work (Rogue et al. 2012). Following treatment with prototypical inducers, HepaRG cells provided E_max_ and EC_50_ values similar to those reported previously (Tables 2, 3) (Grime et al. 2010; Vermet et al. 2016). Likewise, the induced mRNA data were in agreement with effects observed at the enzymatic level (Fig. 1, 2, 3).

A second aim was to determine if the PXR‐KO cells were capable of defining PXR contribution for *CYP3A4* and *CYP2B6* induction. Selective PXR/CAR activators showed a complete loss of *CYP3A4/CYP2B6* induction in the PXR‐KO cells, respectively (Figs. 1, 2), whereas the dual PXR/CAR inducers retained some CYP induction. EC_50_ values for carbamazepine were 38 and 49 *μ*mol/L, for *CYP3A4* in the 5F and PXR‐KO cells, respectively. While the EC_50_ values were similar between cell lines, the *E*_max_ values were ~four‐fold lower in the PXR‐KO cells indicating *CYP3A4* induction is regulated by PXR in addition to another NHR. The same trend was observed for *CYP2B6* and the additional PXR/CAR activator phenytoin. A strong dose–response was observed for *CYP3A4* and *CYP2B2* in the 5F cells following artemisinin treatment. However, in contrast to previous reports (Hariparsad et al. 2008), the inductive effect was abolished for *CYP3A4* in the PXR‐KO cells, indicating artemisinin is a selective PXR activator for *CYP3A4* and a dual PXR/CAR activator for *CYP2B6*.

Interestingly, the selective CAR activator (CITCO) produced a similar *E*_max_, but lower EC_50_ in the PXR‐KO cells compared to other cell types (Fig. 2). The finding could be attributed to compensatory upregulation of other genes, such as the PXR co‐activators p300, CBP and SRC, or the increased expression of additional NHRs. These data are in agreement with HepaRG CAR‐KO activity data (Li et al. 2015). The effect of PB and CITCO has recently been investigated in HepaRG CAR‐KO cells (Li et al. 2015). Treatment with PB and CITCO significantly influenced numerous genes potentially contributing to the EC_50_ fold increase observed in the PXR‐KO cells. Further work is required to fully understand the downstream effect of the gene KO.

Visual inspection of the *CYP2B6* concentration–response curves suggests a biphasic response for CITCO (Fig. 2C and E). Multiple EC_50_ values are possible if the compound can bind with varying affinities to alternative targets on the NHR. This may be due to localization and sequestration of the NHR (Honma et al. 2010) or the involvement of additional NHRs such as the glucocorticoid receptor or the peroxisome proliferator‐activated receptor gamma, but further work is required to understand this process.

In conclusion, this PXR‐KO cell line is a useful novel tool to identify drug–NHR specificity and elucidate the mechanism by which potential DDIs may occur. Utilization of this cell line will allow advancement in composing structure activity relationships rather than relying predominantly on pharmacological manipulations and provide in‐depth mechanistic evaluation.

## Authorship Contributions

Williamson, Mitchell, and Riley participated in research design. Williamson, Lorbeer, and Brayman conducted experiments. Brayman and Mitchell contributed to new reagents or analytic tools. Williamson, Lorbeer, Brayman, and Riley performed data analysis. Williamson, Mitchell, Brayman, and Riley wrote or contributed to the writing of the manuscript.

## Disclosure

None declared.
